# Plasticity in nodal root elongation through the hardpan triggered by rewatering during soil moisture fluctuation stress in rice

**DOI:** 10.1038/s41598-018-22809-5

**Published:** 2018-03-12

**Authors:** Roel Rodriguez Suralta, Jonathan Manito Niones, Mana Kano-Nakata, Thiem Thi Tran, Shiro Mitsuya, Akira Yamauchi

**Affiliations:** 1 Graduate School of Bioagricultural Sciences, Nagoya University, Nagoya, 464 8601 Japan; 2 Crop Biotechnology Center, Philippine Rice Research Institute, Maligaya, Science City of Muñoz, Nueva Ecija 3119 Philippines; 3 International Cooperation Center for Agricultural Education, Nagoya University, Nagoya, 464 8601 Japan; 4 Faculty of Agronomy, Vietnam National University of Agriculture, Trauquy, Gialam, Hanoi Vietnam

## Abstract

Rainfed lowland (RFL) rice fields have hardpans and experience soil moisture fluctuations (SMF) stress, which influence root system development. Here, we clarify the expression and timing of the plasticity in nodal root elongation through the hardpan under SMF and its contribution to shoot growth using a shallow-rooting IR64 and its deep-rooting introgression line, YTH304. Under SMF, soil moisture content had negative relationship with soil penetration resistance, regardless of hardpan bulk densities. YTH304 had greater root system below the hardpan than IR64 in hardpan with 1.50 but not in 1.70 g cm^−3^ bulk density (BD). YTH304 had greater plasticity in nodal root elongation through the hardpan than IR64 under SMF, which was clearly expressed during rewatering. YTH304 also had greater soil water uptake below the hardpan during drought and greater shoot growth than IR64. The results imply that deep root system development during SMF was due to the plasticity in nodal root elongation through the hardpan expressed during rewatering rather than during drought periods. This is against the long standing belief that active root elongation through the hardpan happens during drought. This also implies a need to revisit current root screening methods to identify rice lines with good hardpan penetration ability.

## Introduction

Rice in rainfed lowland (RFL) ecosystems generally yields lower than in irrigated lowlands. This has been conventionally attributed to water stress due to water deficit^1^ or prolonged submergence^2^. In contrast, we have been paying special attention to water stress due to soil moisture fluctuations (SMF)^3,4^. Since RFL is dependent on the availability of rainfall, SMF, which is the recurrence of transiently anaerobic (flooded) to aerobic (mild drought) conditions and vice versa, is a common water stress condition^5–8^. The phenomenon of phenotypic plasticity, defined as the ability of a genotype to change its phenotype in response to changing environmental conditions^9^, has been emphasized as an important crop adaptation strategy to mitigate the effect of stress; thus, maintain productivity^10,11^.

In this aspect, root plasticity is a key trait for plant adaptation to abiotic stresses like water stress^11^. Under SMF, the plasticity in aerenchyma development and lateral root production contributed to the maintenance of root system development under transient drought-to-waterlogged stress condition and vice versa, respectively^3,4,12–15^. Furthermore, the plasticity in root system development based on total root length at the shallow soil layer was shown as important in efficiently capturing the available water after the onset of rainfall^16^. Meanwhile, the ability for deep root system development allowed the access of a greater volume of water from the deeper soil layer during periods of drought^1,17–22^. Both of which have been suggested to improve adaptation to RFL conditions. These two salient traits can either be expressed collectively or independently depending on the kind of particular target RFL rice areas.

A typical RFL rice field has a pronounced hardpan, which is a hardened impervious layer, typically of clay, occurring at varying depths below the soil surface. Hardpan has the highest penetration resistance (PR) along the soil profile^23,24^. It can conserve available water in the shallow soil layer by impairing the drainage during flooded condition (i.e. after occurrence of heavy rainfall), but it also limits root penetration into the deeper soil layer during drought condition^25–27^. Under RFL conditions, the capacity of roots to penetrate hardpan layer is critical for the establishment of deep root system^28^, in which water supply at the shallow layer is more limited^1,29^ than below the hardpan layer^1^ during drought condition. Wax-petrolatum layers is currently used as laboratory method to experimentally simulate the hardpans and screen genotypes with good root penetration ability^26,29–36^. Identified genotypes showed a consistently deep root development in both wax layer screen^34^ and RFL rice field conditions^37^. However, such findings may be due to the inherent constitutive deep root development of those genotypes as shown by the high genotypic correlations in percent nodal root penetration between the 3% (control) and 60% (simulated hardpan) wax layer treatments. Under real RFL rice fields, soil moisture is fluctuating due to the prevailing rainfall patterns. Therefore, the current wax-petrolatum system may not accurately represent the nature of hardpans in RFL because its PR strength is constant regardless of soil moisture conditions.

Soil PR generally interacts with moisture availability^24,38,39^ and the magnitude of soil strength^38–40^. Under this condition, root growth is decreased by both the increase in PR (water is not limiting) and intensity of drought stress (PR is not limiting)^41^. Soil PR may increase more rapidly than decrease in soil moisture^25^; thus, the expression of the plasticity in nodal root elongation through the hardpan in response to drought may be limited. The hardpan PR in RFL rice fields may also influence the expression of root plasticity in response to soil moisture fluctuations.

In general, root system development at deeper soil layer below the hardpan is evident in many RFL rice fields^1,16,24,37^, although genetic variability is small^16,37^. The general notion was that active nodal root elongation through the hardpan happened in response to episodes of drought condition^37^. However, there is still a need to further clarify when and how this process occurred under RFL with fluctuating soil moistures. It is possible that the root penetration through the hardpan during SMF conditions happens when the hardpan PR is relatively low especially during the time when the soil is wet (i.e. after the occurrence of rainfall). Furthermore, the magnitude of hardpan strength may also influence the dynamics of its PR under SMF even when the soil is wet.

Under SMF, we hypothesized that rice genotypes adapted to RFL may have roots that express developmental plasticity triggered by transient soil moisture from dry to wet, which at the same time, would soften the hardpan layer so they can penetrate beyond this layer to access soil water in the deep and contribute to the maintenance of dry matter production. The plasticity in nodal root elongation through the hardpan during rewatering may also be influenced by the magnitude of hardpan strength even when the soil is wet. Thus, this study examined the plasticity and timing of nodal root elongation through the hardpan with varying bulk densities (BD) under SMF, quantify their contribution to water uptake below the hardpan during drought and determine the overall contribution of hardpan-penetrated roots to shoot dry matter production. For this purpose, we used an irrigated lowland variety, IR64 and one of its introgression lines (INL), YTH304. The IR64 (*indica*) is a popular irrigated lowland rice variety in the Philippines. YTH304 is one of the INLs derived from crosses between IR64 and 10 donor varieties including 9 new plant type (NPT) lines and Hoshiaoba (also known as Chugoku146)^42,43^, which yield performance was previously evaluated in aerobic fields. YTH304 was used in this study because of its relatively greater root system development at deeper soil layer and more plastic root branching in response to rewatering in the shallow soil layer than IR64. Thus, it produced more shoot dry matter than its recurrent parent under RFL conditions^16^.

## Results

### Rootbox-hardpan experimental system and soil moisture fluctuation treatments

To examine the plasticity in nodal root elongation through the hardpan under different soil moisture conditions during SMF, we developed a rootbox-hardpan experimental system that can be embedded with 5-cm thick hardpan with variable level of BD at 16 cm below the soil surface (Supplementary Fig. S1). Since the artificial hardpan was made from a mixture of soil and kaolinite, its PR can interact with changes in soil moisture during SMF. This system can also monitor the status of nodal root penetration through the hardpan at different soil moisture conditions during SMF. It can also measure the amount of water taken up by the roots present below the hardpan during progressive drought (DR) period. Two experiments were conducted under greenhouse condition. The first experiment was done to quantify the plasticity and timing of nodal root penetration through the hardpan during SMF. Hardpan BD was set at 1.50 g cm^−3^. The second experiment was done to further examine if such plasticity was affected by varying hardpan BD treatments set at 1.50 and 1.70 g cm^−3^. The two genotypes, IR64 and its INL, YTH304, were grown for 60 and 57 days in Experiment 1 and 2, respectively. These were subjected to well-watered (WW) and SMF conditions.

**Figure 1:**
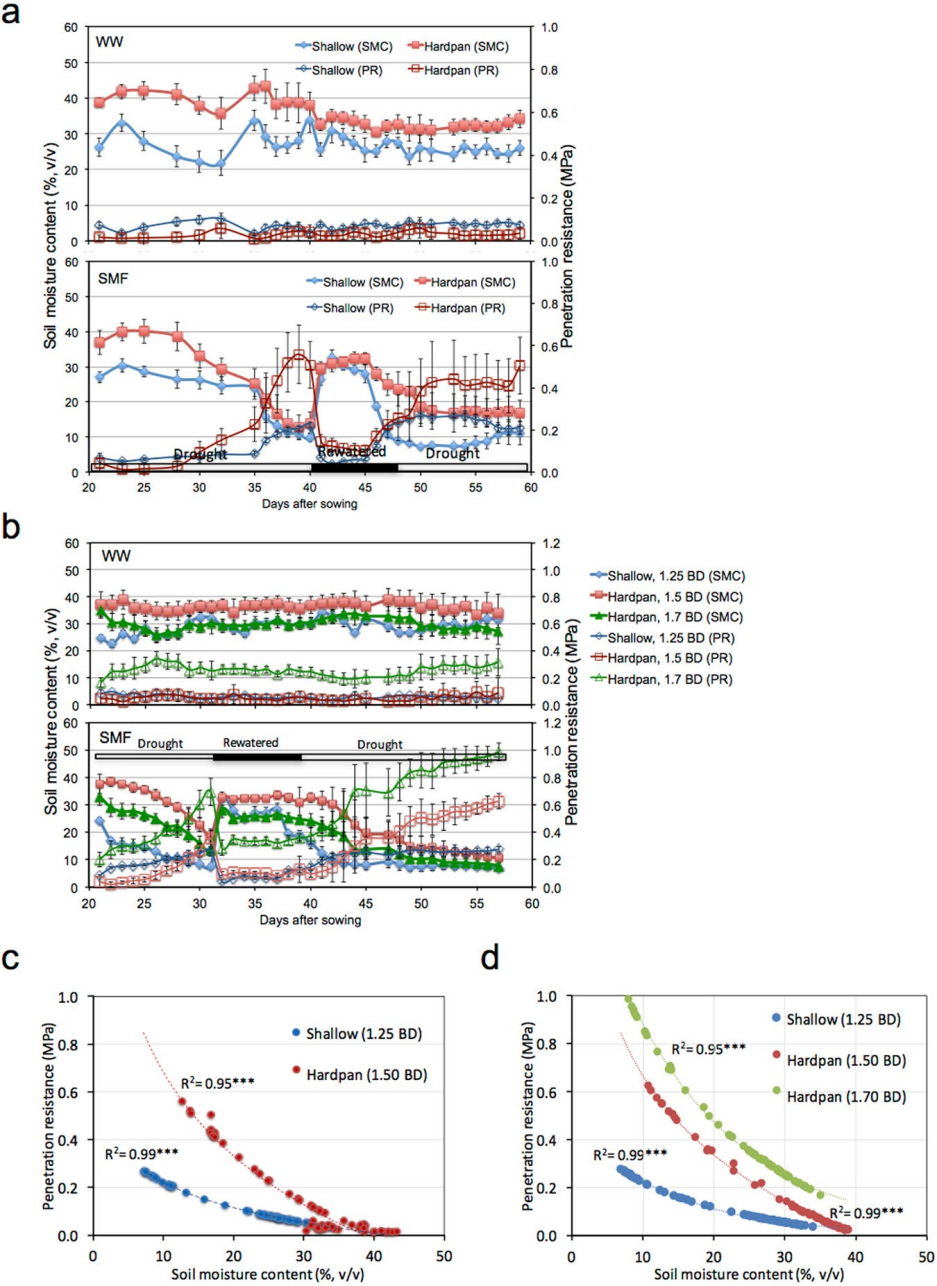
The changes in SMC and PR in shallow and hardpan layers under different water treatments in Exp. 1 (**a**) and under different water and hardpan bulk density (BD) treatments in Exp. 2 (**b**). Horizontal bars along *x*-axis in Fig. 1a,b indicate the prevailing soil moisture conditions during SMF. The relationships between the changes in SMC and PR in shallow and hardpan layers in Exp. 1 and 2, respectively (**c**,**d**). The BD of the shallow layer was 1.25 g cm^−3^ in both Exp. 1 and 2 while that of the hardpan layer was 1.50 g cm^−3^ in Exp. 1 and either 1.50 or 1.70 g cm^−3^ in Exp. 2. WW, well-watered and SMF, soil moisture fluctuation treatments. Error bars represent the standard deviation calculated from 3 replicates. ***Indicate significant at *P* < 0.001.

Evidently, the SMF imposed as episodes of drought-rewatered-drought conditions significantly influenced the soil PR: its strength decreased with increase in soil moisture content (SMC) after rewatering while it increased with decrease in SMC during drought (Fig. 1a,b). The general relationship between changes in SMC and PR was negative and significant (Fig. 1c,d). The PR as well as the magnitude of its increase in response to decreasing soil moisture was higher in hardpan with 1.70 than with 1.50 g cm^−3^ BD (Fig. 1d).

### Plasticity in nodal root elongation through the hardpan and deep root system development under SMF

**Figure 2:**
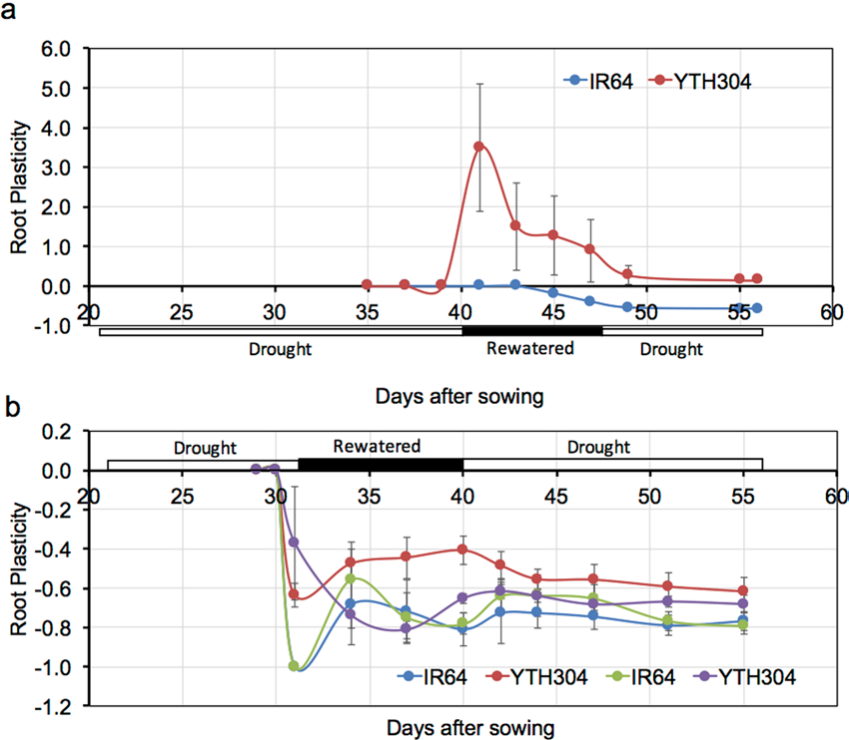
The plasticity in nodal root elongation through the hardpan at different soil moisture conditions during SMF treatment in IR64 and introgression line YTH304 in Exp. 1 (**a**) and Exp. 2 (**b**). The bulk density of the hardpan layer was 1.50 g cm^−3^ in Exp. 1 and either 1.50 (red and blue symbols) or 1.70 g cm^−3^ (violet and yellow green symbols) in Exp. 2. Horizontal bars along *x*-axis indicate the prevailing soil moisture conditions during SMF. Error bars represent the standard deviation calculated from 3 replicates.

We quantified the plasticity and timing of nodal root elongation through the hardpan during SMF. This was done by counting the number of nodal roots that penetrated through the hardpan using a minirhizotron camera inserted at the pre-installed transparent tube directly below the hardpan (Supplementary Figs S1, S2 and S3). The plasticity in nodal root penetration through the hardpan was calculated as the relative change in the number of nodal roots that penetrated the hardpan under SMF compared with WW (control) treatment. The expression in the plasticity in nodal root penetration through the hardpan during SMF was observed during rewatered conditions and was significantly higher in YTH304 than in IR64 (40–47 days after sowing [DAS]) in Exp. 1; Fig. 2a & 32–39 DAS in Exp. 2; Fig. 2b; and Supplementary Figs S2 and S3), especially in 1.50 g cm^−3^ hardpan BD (32–39 DAS in Exp. 2; Fig. 2b and Supplementary Fig. S3). At higher hardpan BD (1.70 g cm^−3^), the plasticity advantage in nodal root penetration through the hardpan during rewatered conditions of YTH304 over that of IR64 was inhibited (32–39 DAS in Exp. 2; Fig. 2b and Supplementary Fig. S3).

**Figure 3:**
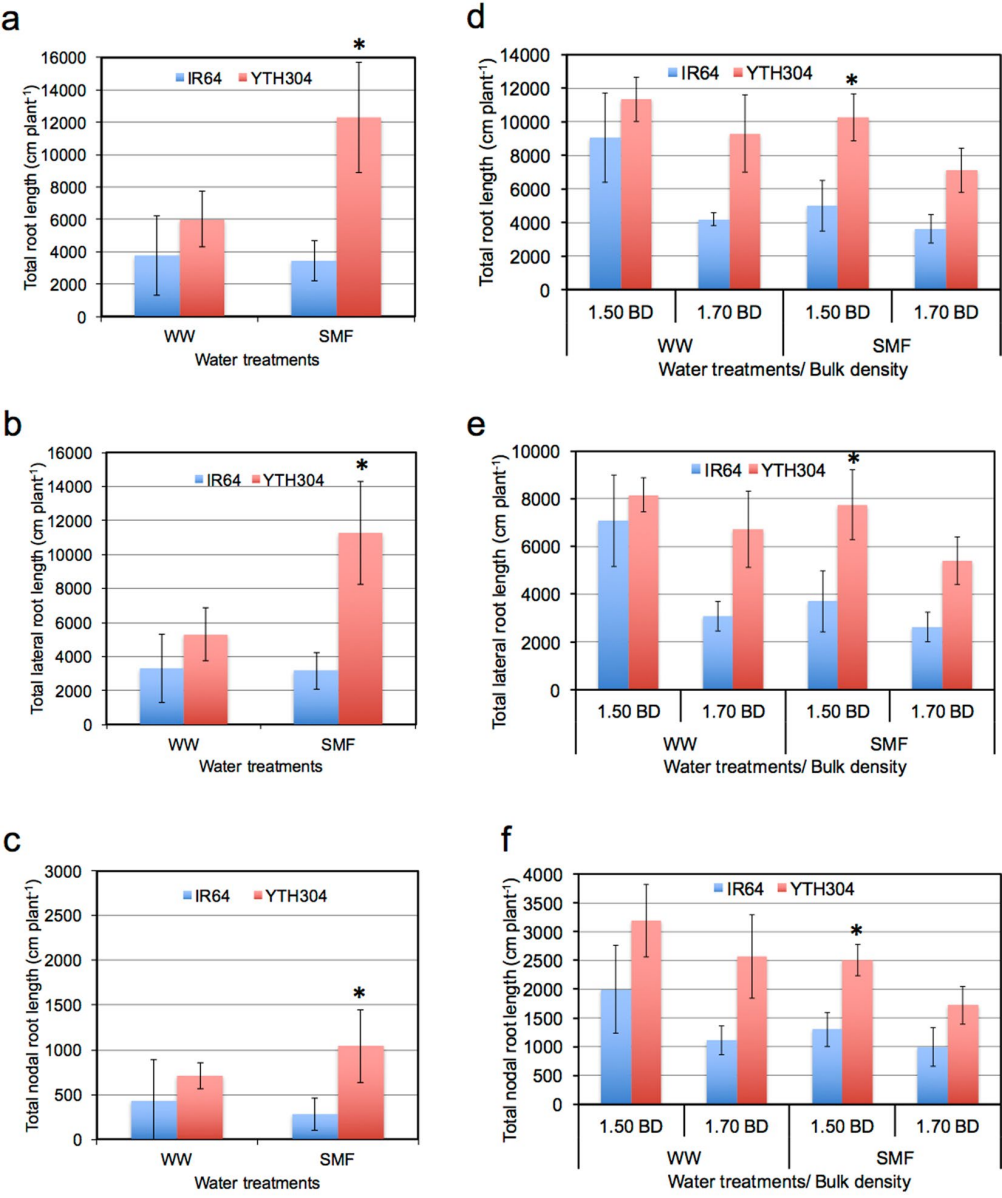
The total root length (**a**,**d**), total lateral root length (**b**,**e**) and total nodal root length (**c**,**f)** below the hardpan layer in IR64 and its INL, YTH304, under different water treatments in Exp. 1 (**a**–**c**) and under different water and hardpan BD treatments in Exp. 2 (**d**–**f**). The bulk density of the shallow soil layer was 1.25 g cm^−3^ while that of the hardpan layer was 1.50 g cm^−3^ in Exp. 1 and either 1.50 or 1.70 g cm^−3^ in Exp. 2. WW, well-watered and SMF, soil moisture fluctuation treatments. Error bars represent the standard deviations calculated from 3 replicates. *Indicates significant difference between the two genotypes at *P* < 0.05, by Student’s t-test.

The SMF also had significant effects on root traits below the hardpan and significant genotypic differences were observed (Fig. 3). We used the relative differences in root traits between YTH304 and IR64 under SMF as an effective measure of plasticity index especially that we were comparing highly genetically similar genotypes with similar shoot and root growth performances under non-stress conditions^44^. In Exp. 1 with 1.5 g cm^−3^ hardpan BD, YTH304 had significantly longer total root length (TRL), total lateral root length (TLRL) and total nodal root length (TNRL) by 345, 367 and 364%, respectively than IR64 under SMF (Fig. 3a–c). Similarly, in Exp. 2, the YTH304 had significantly longer TRL, TLRL and TNRL by 205, 209 and 193%, respectively than IR64 at 1.50 g cm^−3^ hardpan BD (Fig. 3d–f). At higher hardpan BD (1.70 g cm^−3^), these measured root traits were not significantly different between the two genotypes under SMF (Fig. 3d–f). Furthermore, the TRL, TLRL and TNRL above and at the hardpan layers were not significantly different between YTH304 and IR64 regardless of water and hardpan BD treatments (Supplementary Tables 1 and 2).

### Responses in stomatal conductance, water use below the hardpan and overall dry matter production under SMF

**Figure 4:**
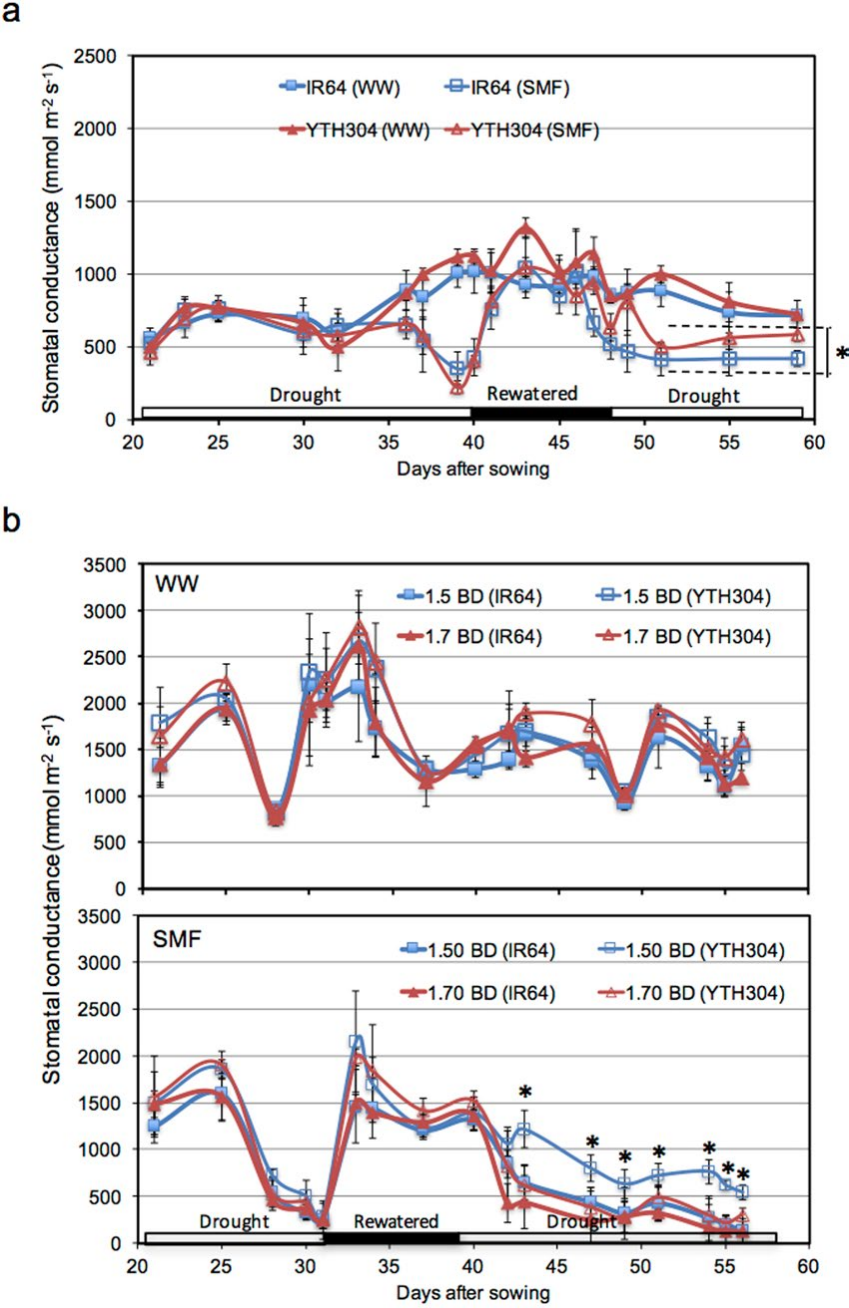
The response of stomatal conductance in IR64 and its INL, YTH304 under different water treatments in Exp. 1 (**a**) and under different water and hardpan bulk density (BD) treatments in Exp. 2 (**b**). Horizontal bars along *x*-axis indicate the prevailing soil moisture conditions during SMF. The BD of the shallow layer was 1.25 g cm^−3^ while that of the hardpan layer was 1.50 g cm^−3^ in Exp. 1 and either 1.50 or 1.70 g cm^−3^ in Exp. 2. WW, well-watered and SMF, soil moisture fluctuation treatments. Error bars represent the standard deviation calculated from 3 replicates. *Indicates significant difference between YTH304 and IR64 under SMF with 1.50 g cm^−3^ BD only at *P* < 0.05, by Student’s t-test.

The changes in stomatal conductance were measured at different soil moisture conditions during SMF. The stomatal conductance was generally reduced in both genotypes during the first drought periods (21–40 DAS in Exp. 1; Fig. 4a & 21–32 DAS in Exp. 2; Fig. 4b). Stomatal conductance under SMF recovered back to the level of their WW controls during rewatered conditions (40–47 DAS in Exp. 1; Fig. 4a & 32–39 DAS in Exp. 2; Fig. 4b). During the first drought and rewatering periods in SMF, the stomatal conductance was similar between genotypes (Fig. 4a,b). However, during the second drought period, stomatal conductance was significantly higher in YTH304 than in IR64, especially in hardpan with 1.50 g cm^−3^ BD (47–57 DAS in Exp. 1; Fig. 4a & 39–57 DAS in Exp. 2; Fig. 4b). At higher hardpan BD (1.70 g cm^−3^), both genotypes had similar stomatal conductance under SMF (Fig. 4b). Under WW, on the other hand, stomatal conductance was similar between genotypes regardless of hardpan BDs (Fig. 4a,b).

**Figure 5:**
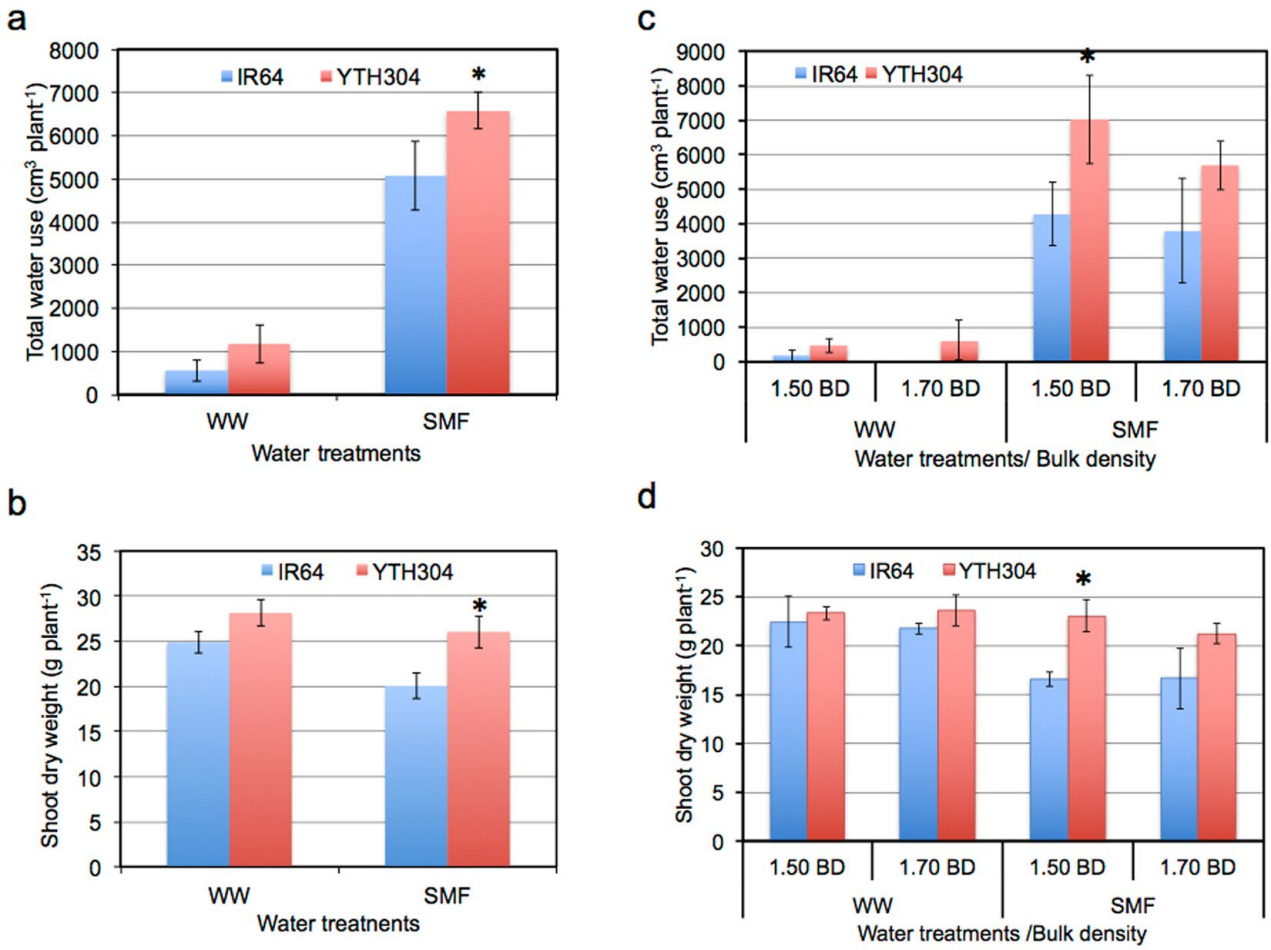
Total water use (**a**,**c**) and shoot dry weight (**b**,**d**) of IR64 and its INL, YTH304, under different water treatments in Exp. 1 (**a**,**b**) and under different water and hardpan bulk density (BD) treatments in Exp. 2 (**c**,**d**). The BD of the shallow soil layer was 1.25 g cm^−3^ while that of the hardpan layer was 1.50 g cm^−3^ in Exp. 1 and either 1.50 or 1.70 g cm^−3^ in Exp. 2. WW, well-watered and SMF, soil moisture fluctuation treatments. Error bars represent the standard deviation calculated from 3 replicates. Asterisk (*) indicates significant difference between the two genotypes at *P* < 0.05, by Student’s t-test.

Water use was measured during DR period in SMF using Mariotte’s bottle technique to examine the capacity of roots (if present) below the hardpan layer to take up water from the deep when the shallow soil layer was drying. Generally, YTH304 had significantly greater root water uptake below the hardpan by 30% in Exp. 1 (Fig. 5a) and 64% in Exp. 2 (Fig. 5c) than IR64 at 1.5 g cm^−3^ hardpan BD under SMF. Consequently, YTH304 produced a significantly greater shoot dry matter production by 30% in Exp. 1 (Fig. 5b) and 39% in Exp. 2 (Fig. 5d) than IR64 at 1.5 g cm^−3^ hardpan BD under SMF. At higher hardpan BD (1.7 g cm^−3^), both genotypes had similar root water uptake below the hardpan and shoot dry matter production (Fig. 5c,d). Under WW, on the other hand, the root water uptake below the hardpan layer and the shoot dry matter production were similar between the two genotypes regardless of hardpan BDs (Fig. 5).

## Discussion

The plasticity of root system development is a key trait for plant adaptation to various types of water stress; thus, there is a need to identify plasticity traits that should be considered in designing a breeding program for a particular abiotic-stressed rice production environment^11^. The plasticity in deep root system development is important for the greater access of soil moisture at depths, especially when the upper soil surface in RFL fields suffers drought. Most of the studies on root growth and penetration ability, however, have focused more on the responses to drying soil conditions^45^ where the PR substantially increases; thereby, inhibiting root growth^23^. The current available laboratory screening systems for root penetration ability under RFL conditions assumes that hardpan PR is constant^33,34^. In reality, however, soil PR under RFL rice fields negatively interacts with changes in soil moisture conditions^23,24^. Recognizing these limitations, we have successfully designed a rootbox-hardpan experimental system (Supplementary Fig. S1) that sets the hardpan at desired BD, which naturally interacts with SMF (Fig. 1). Through this system, we have studied the expression and timing of the nodal root penetration through the hardpan in real time (Fig. 2; Supplementary Figs S2 and S3). It can also quantify the root water uptake below the hardpan during DR period in SMF (Fig. 5a,c).

In this study, YTH304 had significantly greater plasticity in nodal root elongation through the hardpan during rewatered condition, greater root system development and root water uptake below the hardpan, greater stomatal conductance and shoot dry matter production than the recurrent parent IR64 under SMF especially at 1.50 g cm^−3^ hardpan BD. The plasticity in nodal root elongation through the hardpan under SMF at 1.50 g cm^−3^ hardpan BD occurred in two steps. First, the plasticity in nodal root elongation through the hardpan was expressed in response to rewatering period during SMF (Fig. 2a,b). Second, once the succeeding drought occurred at the shallow soil layer, the nodal roots that penetrated through the hardpan continued to elongate and develop greater root system at the deep (Fig. 3). Overall, such root plasticity expression substantially increased root water uptake below the hardpan during period of drought stress (Fig. 5a,c) and contributed to the maintenance of greater shoot dry matter production (Fig. 5b,d). To the best of our knowledge, this is the first report showing that the plasticity in nodal root elongation through the hardpan in rice happened during rewatering rather than during progressive drought under SMF. This supports our previous works that highlighted the importance of root plasticity in maintaining water uptake, dry matter production and yield in water stressed soil environments^3,4,11–13,16,46–49^.

The mechanism for the plasticity in nodal root elongation through the hardpan during SMF (i.e. drought to rewatered condition) is not yet fully understood. Under drought, the genetic^50–52^, physiological^53–56^, anatomical^55,57^ and morphological^58^ bases for root growth are well studied especially in unimpeded soils. When drought occurrence is associated with an increase in soil PR, occurrence of root growth is very limited^41,45^ and may involve additional and/or another mechanism. Rapid elongation rates of previously drought stressed hardpan impeded nodal roots may be associated with the rapid development of relatively long root elongation zones^59^ in response to rewatering and a decrease in PR. This will give an advantage for greater root system development in preparation for the next episode of drought during SMF. However, this will be a subject for further investigations.

Root thickness is considered as an important trait contributing to root penetration ability in strong soils. An increase in thickness of impeded roots in drought-stressed soil or strong wax layers is caused by cortical cells enlarging radially rather than axially, with a corresponding change in the orientation of the cellulose microfibrils in the cell walls^60^. The thickening of the roots may relieve stress in front of the root apex and decrease buckling in drying hard soils^61^. In rice, genotypes that produced longer root lengths under strong wax layers had also thicker roots^26^. However, these same set of genotypes also produced greater root lengths with or without wax layers, although they had reduced root lengths and increased root thickness in with wax layer. This may indicate that deep penetration ability under strong soils was influenced by the inherent ability of genotypes to develop roots at deeper layer regardless of soil strengths. In maize, stele diameter contributed to root tensile strength than root diameter per se^62^. Thus, root thickness may have nothing to do with hardpan penetration ability as thick roots do not necessarily signify strong roots. Instead, the root tip geometry may be a better indicator of root penetration ability in soils of greater strength^41,63^.

The expression of the plasticity in nodal root elongation through the hardpan during rewatered conditions under SMF was also dependent on the magnitude of hardpan BD. Thus, the increase in hardpan BD limited the expression of the plasticity in nodal root elongation through the hardpan (Fig. 2b), root system development (Fig. 3d) and water uptake (Fig. 5c) below the hardpan, stomatal conductance (Fig. 4b) and shoot dry matter production (Fig. 5d) as indicated by the similar values for such traits between the two genotypes at 1.70 g cm^−3^ hardpan BD. At such hardpan BD, the PR was already higher than that of hardpan with lower BD (1.50 g cm^−3^) despite the presence of higher soil moisture. The corresponding increase in PR during DR periods in SMF was also higher than in hardpan with lower BD (1.50 g cm^−3^; Fig. 1b). The inhibition of the expression of root plasticity with an increase in hardpan BD can be attributed to the very low soil moisture (10% SMC, v/v) as drought progressed during SMF, which may be enough to cause severe stress to the roots and inhibits the expression of root plasticity in terms of faster resumption of growth during rewatering^46^. It is also possible that the expression of plasticity in nodal root elongation through the hardpan of YTH304 under SMF is also limited only to a hardpan with moderate BD (1.50 g cm^−3^). Hence, the present results need to be validated using other genetic backgrounds under greenhouse and field conditions. The study may also identify other genotypes, which may have greater plasticity in nodal root elongation through the hardpan during rewatering even at higher hardpan BDs.

The present study provides a starting point to further understand the real target RFL rice field environments for which rice varieties are being developed to increase yield. There is a possibility that the expression in the plasticity in nodal root elongation through the hardpan may not work in hardpan with very high PR. The maximum soil PR measured in this study was around 1 MPa at 1.70 g cm^−3^ BD. On the other hand, those that used wax-layer to simulate the soil hardpan with PR ranged from 1 to 1.50 MPa^26,30,32,33^ showed large genotypic variations in terms of root penetration ability^26,30,32,33^. These experimentally tested values are generally lower than the measured soil penetration values in real RFL fields. The soil PR at 20–30 cm depths in various lowland rice sites across South and South-East Asia ranged from 1.02 to 5.73 MPa in fields with standing water while it ranged from 2.35 to 6.88 MPa in fields without standing water^23^. Majority of these areas had soil PR at 20–30 cm depth greater than 3.0 MPa regardless of soil water status^23^. This may show that rice roots cannot penetrate hardpans under real rainfed lowlands even when the soil is fully saturated and more so when the soil is drying. In the absence of the expression of the plasticity in nodal root elongation through the hardpan in response to rewatering under RFL rice with strong hardpans, the plasticity in root system development at the shallow layer above the hardpan is also equally important in contributing to the promotion in shoot dry matter production^16^ and yield. South and Southeast Asian rice fields have large variations in hardpan PR^23^, which suggests that the expression of root plasticity under RFL rice conditions is governed by the genotype by environment interactions.

Aside from wide variations in PR among RFLs sites^23^, the pattern of subsoil resistance with soil depths also differed^23,33,64^. There is either a distinct hardpan similar to the ones used in this study, or no hardpan in which soil strength increases steadily with depths^23,65^. Such differences in the pattern of subsoil compactions also influenced the root penetration ability even under constant moisture environments in both rice^35^ and wheat^64^.

In many soils, series of cracks and biophores in the hardpan that appear as a consequence of soil drying^45,65^ could also influence nodal root penetration during rewatered conditions. Rewetting may fill large cracks in the hardpan with soft top soil, which may also provide a soft space for nodal roots to avoid high impedance in reaching the deep soil layers. Thus, there is a need to consider different strengths of hardpan and pattern of PRs with soil depth and degree of soil cracking in relation to SMF to elucidate further our understanding of the root plasticity expressions, as well as hydrologic patterns under RFL conditions. This will further enhance our knowledge in pinpointing the kind of root ideotype custom-tailored to the nature of the target RFL environments, as well as properly design the corresponding high throughput screening techniques.

## Methods

Two experiments were conducted in a greenhouse of the Graduate School of Bioagricultural Sciences, Nagoya University, Japan (136° 56′ 6′′ E, 35^o^ 9′ 5′′ N) during the summer months of 2013 and 2014.

### Plant cultivations

The seeds of IR64 and its INL, YTH304 were soaked in water and incubated in a seed germinator maintained at 28 °C for 24 h prior to sowing. Three pre-germinated seeds from each genotype were grown in a rootbox at two hills per box. The seedlings were later thinned to one seedling per hill at 3 DAS. The plants were grown for 60 days in Experiment 1, and 57 days in Experiment 2. At 21 DAS prior to the first drought initiation, the top of each rootbox was covered with aluminum foil leaving only the plants exposed outside to minimize soil water evaporation.

### Rootbox-hardpan experimental system

The rootboxes were made of opaque polyvinyl chloride with 5 mm thickness (Supplementary Fig. S1). The box dimension was 34 cm in length, 9.5 cm in width and 64 cm in depth that constituted the effective inner space. Except for the removable sidewall (35 cm × 65 cm), the other sides and the bottom were bound together with extra strength bonding agent. The removable sidewall was bound tightly to the box with a rubberized sealant. At 31 cm below the top of the box, a transparent tube (7 cm in inner diameter and 40 cm in length) was installed and bound with a rubberized sealant. This transparent tube was positioned just below the artificial hardpan layer where the mini-rhizotron camera was inserted to monitor the development of the penetrated nodal roots during different timings of SMF. The transparent tubes were covered on both ends with plastic cups wrapped with aluminum foil.

Small plastic connector was installed below where the hardpan soil layer (55 cm below the soil surface) was embedded. This tube, placed on one of the narrow sides of each box, served to connect the box and the graduated cylinder. The top end of the graduated cylinder was sealed with a rubber stopper with plastic tubes inserted on it, which is in accordance to the principle of Mariotte’s bottle.

### Experiment 1. Evaluation of the timing and plasticity of nodal root elongation through the hardpan during soil moisture fluctuations

In this experiment, we initially examined if there was plasticity in nodal root elongation through the hardpan and the timing of its expression during SMF. Hardpan BD was set only at 1.50 g cm^−3^ across two soil water treatments namely, WW (control) and SMF as stress conditions. In WW, SMC was maintained to at least 34% (v/v), by regular watering at the shallow surface. In SMF, the soil was maintained similar to that of the WW control from 0 to 21 DAS, then exposed to DR from 21 DAS until SMC of the shallow soil layer reached down to 10%, which in this experiment, happened at 40 DAS. The target 10% SMC was close to the critical soil moisture (8% SMC or −0.28 MPa) when most legumes and cereals we have tested started showing sign of wilting. This happened especially in the late afternoon, but recovered the next morning. After progressive DR, rewatering was done and maintained at WW condition from 40 to 47 DAS. Finally, DR was again imposed from 47 DAS until the SMC of the shallow soil layer reached down to 10% and maintained to that level of SMC until 57 DAS. The SMCs at soil surface (0–12 cm) were measured using the time domain reflectometry probe (TDR; Tektronix Inc., Wilsonville, OR, USA). Two stainless steel rods (15 cm in length and 3 cm apart) were inserted into the soil at a depth of 12 cm, allowing a 3 cm protruding above the soil surface where TDR probes were attached to obtain SMC readings^66^.

### Experiment 2. Evaluation of the plasticity and timing of nodal root elongation through the hardpan during soil moisture fluctuations as affected by hardpan bulk density

In this experiment, we examined if the plasticity in nodal root penetration through the hardpan is affected by the hardpan’s BD. Thus, we used two hardpan bulk density treatments: 1.50 and 1.70 g cm^−3^ and subjected to WW and SMF conditions. The WW was imposed as described above. On the other hand, the SMF treatment was first maintained under WW conditions from 0 to 21 DAS, then managed with DR from 21 DAS until the SMC reached down to 10%, which happened at 32 DAS. Thereafter, rewatering was done and maintained at WW condition from 32 to 39 DAS. Finally, the plants were subjected again to DR from 39 DAS until the SMC reached down to 10% and maintained to that level of SMC until 57 DAS.

### Soil and bulk density preparations, and rate of fertilizer applications

The air-dried sandy loam soil, sieved through a 3-mm screen, was mixed thoroughly with compound fertilizer (14-14-14) at the rate of 60 mg kg^−1^ soil. Each box was divided into three layers: shallow (0–16 cm), hardpan (16–21 cm) and below the hardpan (21 to 63 cm below the soil surface) layers. The portion corresponding below the hardpan layer was filled up first with 15.55 kg soil to achieve a bulk density at 1.25 g cm^−3^. For the creation of soil hardpan, a mixture of 95% soil and 5% kaolinite (Al_2_Si_2_O_5_(OH)_4_, 0.5% moisture content, pH 4.3 and 0.29 g cm^−3^ specific gravity) was used. The 2.422 kg soil-kaolinite mixture was first partially wetted and then slowly filled into the 5-cm layer (16 to 21 cm below the soil surface). This was followed by repeated manual compaction using a heavy iron bar (10 cm × 8 cm × 50 cm, L × W × H) to achieve a BD of 1.50 g cm^−3^ for Experiment 1. In Experiment 2, the hardpan layer was set to a BD at either 1.50 g cm^−3^ (as described above), or 1.70 g cm^−3^. The latter hardpan BD was achieved by slowly filling up with partially wetted soil (2.74 kg) premixed with 45% silica sand and 10% kaolinite into the 5-cm layer in the rootbox. As described above, this was followed by repeated manual compaction using a heavy iron bar. Finally, the shallow layer was filled with 6.46 kg soil to achieve BD (1.25 g cm^−3^) similar to that of the soil below the hardpan layer. Two TDR stainless rods (12 cm in length) were inserted at 3 cm apart at the surface of the shallow soil layer and on the side of the rootbox parallel to the hardpan soil layer. These stainless rods were used to monitor the SMCs of the two soil layers using TDR meter.

### Stomatal conductance measurements

The stomatal conductance at different periods of soil moisture conditions during SMF was measured on the second youngest fully developed leaf of each plant using a steady state diffusion leaf porometer (SC-1 Leaf Porometer, Decagon Devices Inc., USA) at 1000 h.

### Soil moisture content and penetration resistance measurements

The SMC (%, v/v) was monitored using a TDR meter attached to the pre-inserted TDR nails in the shallow and hardpan soil layers. The soil PR (MPa) for different soil BDs was estimated using the following relationships generated from an earlier small pot experiments (Supplementary Method 1 and Supplementary Fig. S6).

1 $$1.25\,{\rm{g}}\,{{\rm{cm}}}^{-3}\,{\rm{soil}}\,{\rm{bulk}}\,\mathrm{density},\,{\rm{SPR}}=-0.156\,\mathrm{ln}({\rm{SMC}})+0.5871$$

2 $$1.50{\rm{g}}\,{{\rm{cm}}}^{-3}\,{\rm{soil}}\,{\rm{bulk}}\,\mathrm{density},\,{\rm{SPR}}=-0.377\,\mathrm{ln}({\rm{SMC}})+1.701$$

3 $$1.70\,{\rm{g}}\,{{\rm{cm}}}^{-3}\,{\rm{soil}}\,{\rm{bulk}}\,{\rm{density}},\,{\rm{SPR}}=-0.549\,\mathrm{ln}({\rm{SMC}})+2.121$$

where SPR is the soil penetration resistance (MPa) and SMC is soil moisture content (%, v/v).

### Monitoring and counting of hardpan penetrated nodal roots

Monitoring of the roots penetrating the hardpan at different timings during soil moisture fluctuation treatments was done using a mini rhizotron camera (Regents Instruments Inc., USA) inserted to pre-installed transparent tubes in each box. The images were scanned and stored in the computer. The number of penetrated nodal roots visually observed from each image was counted. Root plasticity in nodal root elongation through the hardpan was defined as the number of nodal roots that penetrated through the hardpan under stress treatment compared with the control conditions. This was calculated using single replicates from the SMF (stress) treatment and mean values from the WW (control) treatment:

4 $$\mathrm{Root}\,\mathrm{plasticity}=[\frac{{\rm{Xstress}}-\bar{{\rm{X}}}\mathrm{control}}{\bar{{\rm{X}}}\mathrm{control}}]$$

### Measurement of root water uptake below the hardpan layer

The water uptake at the deep of hardpan-penetrated roots was monitored during the second period of DR under SMF using the attached graduated cylinders (Supplementary Fig. S1) following the principle of Mariotte’s bottle. The amount of water reduced from the graduated cylinder is equivalent to the amount of water absorbed by the roots below the hardpan soil layer especially during the period of DR. The amount of water reduced inside the graduated cylinder was replenished whenever necessary.

### Shoot and root trait measurements

Plant samplings were done at the termination of each experiment. The number of tillers of each plant was counted, cut then oven dried at 80 °C for 48 h before weighing.

The removable sidewall panel on one side of each box was carefully removed, and then the roots were carefully extracted in the following order: below the hardpan, hardpan and shallow soil layers. The roots were obtained by spraying with water to remove soil. Thereafter, the root system from each soil layer was stored in FAA (formalin: acetic acid: 70% ethanol = 1: 1: 18 by volume) solution for processing and measurements. The total number of nodal roots at the base of each plant was manually counted. For TRL, root samples from each soil layer was spread in a transparent container with thin layer of water and then scanned at 600 dpi (EPSON Expression 10000XL). Scanned images were analyzed for root length using WinRhizo v. 2007d (Régent Instruments, Québec, Canada) with a pixel threshold value set at 175. The TLRL was estimated as the total length of roots with less than ≤ 0.2 mm in diameter. The TNRL is the difference between the TRL and TLRL.

Experiment 1 was arranged in split plot design in RCBD with water treatments assigned as mainplots while the genotypes as subplots. Experiment 2 was arranged in split split-split plot design in RCBD with water treatments assigned as mainplots, hardpan BD as subplots and genotypes as sub-subplots. All experimental treatments were replicated 3 times. The ANOVA and calculation of means were done for all measured traits using IRRISTAT program (version 4.1). The mean differences between genotypes under each water or BD treatment were determined by Student’s t-test at *P* < 0.05.

## Electronic supplementary material

Supplementary Information
