# Supplementary material for: Plasticity in nodal root elongation through the hardpan triggered by rewatering during soil moisture fluctuation stress in rice
Source: Sci Rep. 2018 Mar 12;8:4341. doi: 10.1038/s41598-018-22809-5 (PMC5847610; doi:10.1038/s41598-018-22809-5)
Supplement: Supplementary file 1 — Supplementary Information [file 41598_2018_22809_MOESM1_ESM.docx]

**Supplementary Information**

**Plasticity in nodal root elongation through the hardpan triggered by rewatering during soil moisture fluctuation stress in rice**

Roel Rodriguez Suralta, Jonathan Manito Niones, Mana Kano-Nakata, Thiem Thi Tran, Shiro Mitsuya and Akira Yamauchi

**Supplementary Method 1**. Calibration between the changes in soil moisture content and penetration resistance under progressive drought stress

An experiment was established to determine the relationship between the changes in moisture content and penetration resistance. Air-dried sandy loam soil was sieved though a 3 mm mesh and packed in plastic pots (11 cm diameter x 12.5 cm height) at three dry soil bulk densities: 1.25 (light compaction), 1.50 g cm^-3^ (medium compaction) and 1.7 g cm^-3^ (heavy compaction). Thus, three sets of pots (3 pots per set) were filled with 1484, 1781 and 2018 g soil pot^-1^ for lightly, medium and heavily compacted soil treatments, respectively. The lightly compacted treatment was filled with pure soil only. The medium compacted soil consist of 95% soil mixed with 5% kaolinite ([Al](http://en.wikipedia.org/wiki/Aluminium)_2_[Si](http://en.wikipedia.org/wiki/Silicon)_2_[O](http://en.wikipedia.org/wiki/Oxygen)_5_([OH](http://en.wikipedia.org/wiki/Hydroxide))_4_, 0.5% moisture content, 4.3 pH and 0.29 g ml^-1^ specific gravity). The heavily compacted soil, which consists of 45% soil, 45% silica sand, and 10% kaolinite. Two stainless nails (15 cm length) nails at 3 cm apart were inserted at 12 cm depth into the soil. The pots were initially filled with water up to flooded conditions and then watering was withheld to allow progressive soil drying. The soil moisture contents (SMC [% v/v]) were measured by attaching the time-domain reflectometry (TDR; Tektronix Inc., Wilsonville, OR, USA) soil moisture meter to the pre-inserted stainless nails as described above. The soil penetration resistance was measured by pushing an E280 pocket penetrometer (Geotest Instrument Corp., Burr Ridge, IL 60528, USA) into the soil. The measurements were done daily during the duration of progressive drought stress treatment. Correlation analysis between SMC and soil penetration resistance were analyzed using Pearson two-sided correlation analysis in R. The relationship is presented in Supplementary Fig. S6.

**

**Supplementary Figure S1.** Graphical illustration of the rootbox-hardpan experimental system.


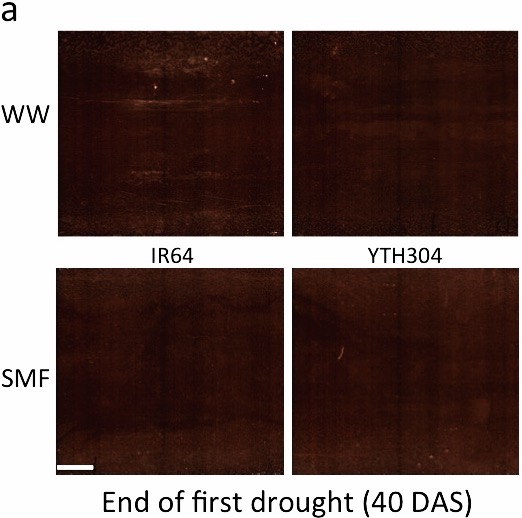

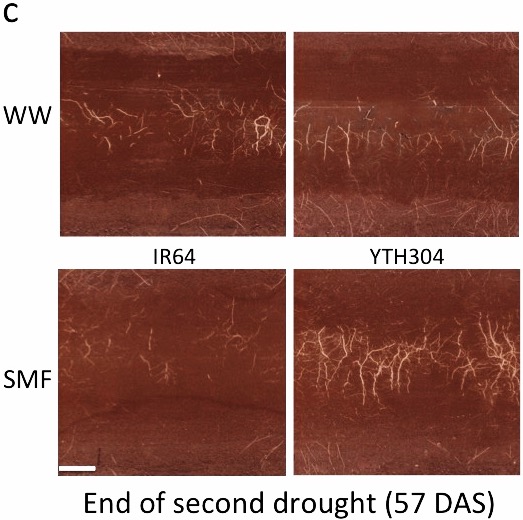


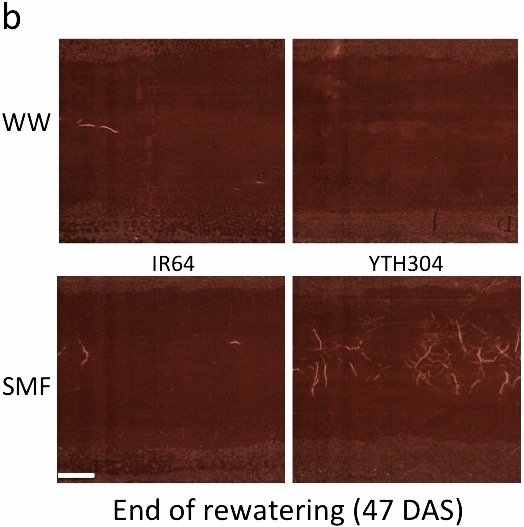


**Supplementary Figure S2** (a-c) Visual observations of the hardpan penetrated nodal roots using a Minirhizotron camera at different soil moisture conditions during soil moisture fluctuation (SMF) treatment in IR64 and introgression line YTH304 under different water treatments (Exp. 1). White bars= 5 cm. (d) The number of nodal roots below the hardpan of each genotype under different water treatments. The bulk density of the shallow soil layer was 1.25 g cm^-3^ while that of the hardpan layer was 1.50 g cm^-3^ in all water treatments. Error bars represent the standard deviation calculated from 3 replicates. Horizontal bars along *x*-axis in Fig. 2d indicate the soil moisture conditions during SMF. Asterisk (*) indicates significant differences between the two genotypes under SMF only at *P*<0.05, by Student’s t-test. WW, well-watered; SMF, soil moisture fluctuation treatments.

a b


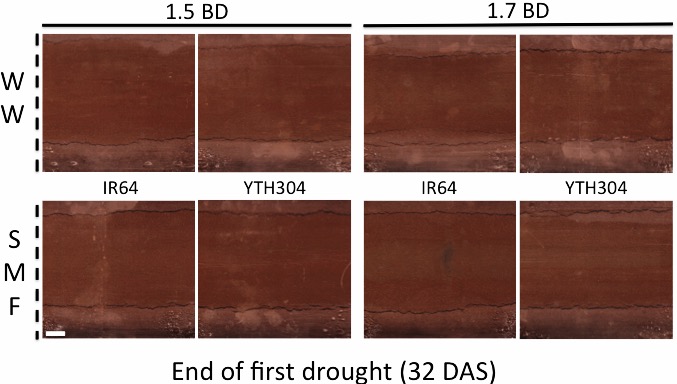

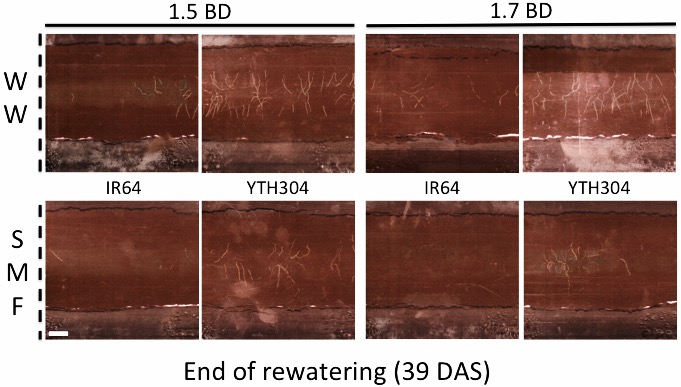


c d


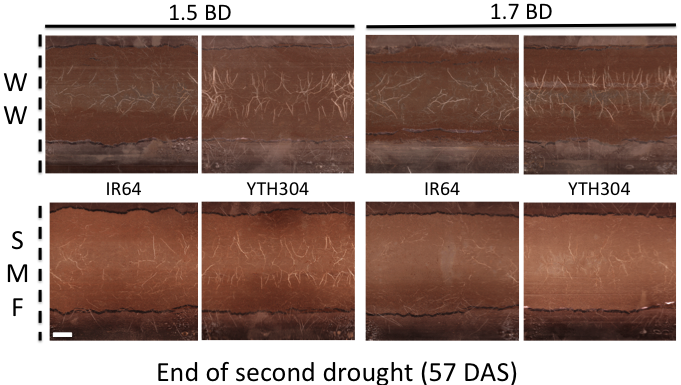


**Supplementary Figure S3** Visual observations of the hard penetrated nodal roots using a Minirhizotron camera at different soil moisture conditions during soil moisture fluctuation (SMF) in IR64 and its introgression line YTH304 under different hardpan bulk densities (BD) and water treatments (Exp. 2). White bars= 5 cm. (d) The number of hardpan penetrated nodal roots of each genotype under different BD treatments. The BD of the shallow layer was 1.25 g cm^-3^ while that of the hardpan layer was either 1.50 or 1.70 g cm^-3^. Error bars represent the standard deviation calculated from 3 replicates. Horizontal bars along *x*-axis indicate the soil moisture conditions during SMF. Asterisk (*) indicates significant difference between the two genotypes at certain days under each BD treatment at *P*<0.05, by Student’s t-test. WW, well-watered; SMF, soil moisture fluctuation treatments.

**Supplementary Fig. S4.** Root system development below the hardpan layer in IR64 and its introgression line YTH304 under different water treatments (Exp. 1). White bars= 5 cm. The soil bulk density of the shallow soil layer was 1.25 g cm^-3^ while that of the hardpan soil layer was 1.50 g cm^-3^ regardless of water treatments. WW, well-watered; SMF, soil moisture fluctuation treatments.


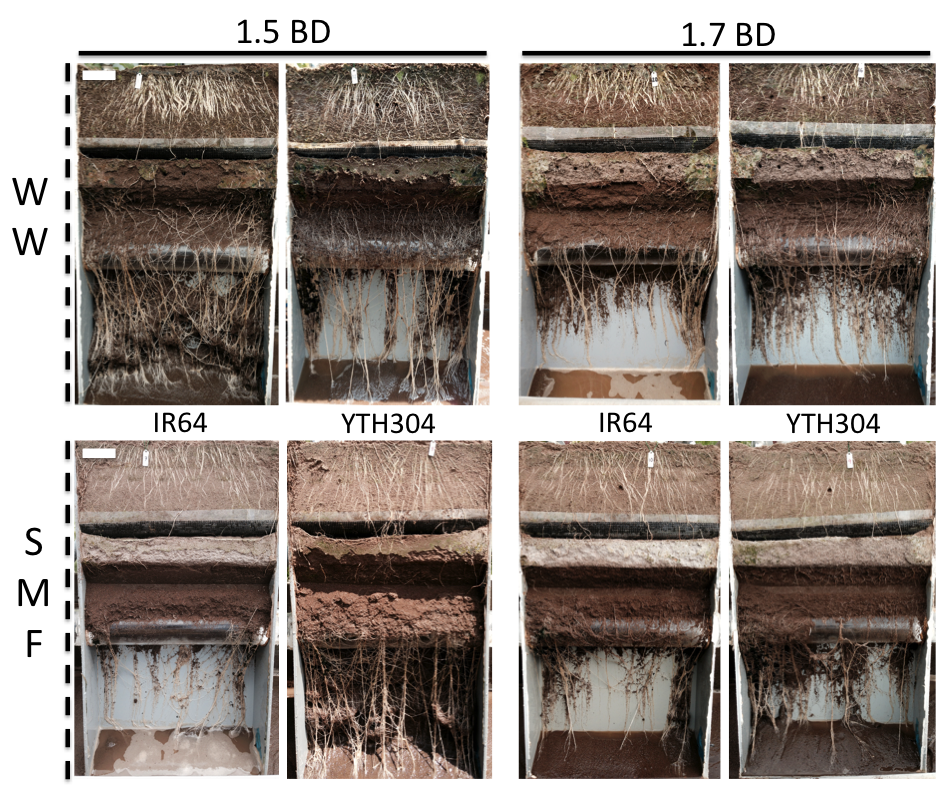


**Supplementary Figure S5** Root system development below the hardpan layer in IR64 and its introgression line YTH304 under different hardpan bulk densities (BD) and water treatments (Exp. 2). White bars= 5 cm. The BD of the shallow soil layer was 1.25 g cm^-3^ while the hardpan layer was either 1.50 or 1.70 g cm^-3^. WW, well-watered; SMF, soil moisture fluctuation treatments.

**Supplementary Figure S6.** The relationship between soil moisture content (% v/v) and soil penetration resistance (MPa) under different soil bulk densities (g cm^-3^). ***, significant at *P*<0.001.

**Supplementary Table 1**. Number of tillers per plant and component root traits of IR64 and its introgression line YTH304 under well-watered (WW) and soil moisture fluctuation (SMF) conditions.

| Soil layer | Water treatment | Genotypes | Tillers  (no. plant^-1^) | Nodal roots (no. plant^-1^) | | Total root length  (cm) | Total nodal root length (cm) | Total lateral root length (cm) |
| --- | --- | --- | --- | --- | --- | --- | --- | --- |
| Shallow | WW | IR64 | 28.8 | 233.3 | 20889.3 | | 3720.0 | 17169.3 |
|  |  | YTH304 | 27.5^ns^ | 212.0^ns^ | 21807.5^ns^ | | 3794.3^ns^ | 18013.2^ns^ |
|  | SMF | IR64 | 32.8 | 227.8 | 15405.2 | | 2782.9 | 12622.9 |
|  |  | YTH304 | 31.5^ns^ | 224.7^ns^ | 17538.7^ns^ | | 3788.1^ns^ | 13750.6^ns^ |
| Hardpan | WW | IR64 |  |  | 5866.1 | | 912.7 | 4953.4 |
|  |  | YTH304 |  |  | 7793.0^ns^ | | 1174.5^ns^ | 6618.6^ns^ |
|  | SMF | IR64 |  |  | 6157.8 | | 784.7 | 5373.1 |
|  |  | YTH304 |  |  | 6302.4^ns^ | | 894.2^ns^ | 5408.2^ns^ |

ns, not significantly different between genotypes under each water treatment and soil layer

**Supplementary Table 2.** Number of tillers per plant and component root traits of IR64 and its introgression line YTH304 under different water and hardpan bulk density treatments (Exp. 2). Bulk density (BD): 1.50 and 1.70 g cm^-3^ soil. Water treatments: well-watered (WW) and soil moisture fluctuation (SMF) conditions.

| Soil layer | Water treatment | Hardpan bulk density (g cm^-3^) | Genotype | Tillers  (no. plant^-1^) | Nodal roots (no. plant-1) | Total root length  (cm) | Total nodal root length (cm) | Total lateral root length (cm) |
| --- | --- | --- | --- | --- | --- | --- | --- | --- |
| Shallow | WW | 1.50 | IR64 | 17.3 | 270.8 | 35646.6 | 2887.0 | 32759.6 |
|  |  |  | YTH304 | 15.2^ns^ | 250.3^ns^ | 37834.6^ns^ | 2267.9^ns^ | 35566.7^ns^ |
|  |  | 1.70 | IR64 | 16.2 | 210.5 | 37220.8 | 2293.4 | 34927.3 |
|  |  |  | YTH304 | 14.5^ns^ | 229.5^ns^ | 39159.8^ns^ | 1758.3^ns^ | 37401.5^ns^ |
|  | SMF | 1.50 | IR64 | 17.3 | 149.5 | 22448.4 | 1685.5 | 20762.9 |
|  |  |  | YTH304 | 20.2^ns^ | 183.2^ns^ | 19365.6^ns^ | 1559.6^ns^ | 17805.9^ns^ |
|  |  | 1.70 | IR64 | 18.3 | 184.7 | 21706.6 | 1418.9 | 20287.8 |
|  |  |  | YTH304 | 17.2^ns^ | 175.8^ns^ | 22769.7^ns^ | 1649.3^ns^ | 21052.3^ns^ |
| Hardpan | WW | 1.50 | IR64 |  |  | 10027.9 | 596.8 | 9431.0 |
|  |  |  | YTH304 |  |  | 10600.4^ns^ | 747.8^ns^ | 9852.5^ns^ |
|  |  | 1.70 | IR64 |  |  | 9089.7 | 495.0 | 8594.6 |
|  |  |  | YTH304 |  |  | 9227.5^ns^ | 612.8^ns^ | 8614.7^ns^ |
|  | SMF | 1.50 | IR64 |  |  | 6409.8 | 431.5 | 5978.2 |
|  |  |  | YTH304 |  |  | 5489.8^ns^ | 442.8^ns^ | 5047.0^ns^ |
|  |  | 1.70 | IR64 |  |  | 4608.7 | 261.3 | 4347.4 |
|  |  |  | YTH304 |  |  | 5116.6^ns^ | 364.6^ns^ | 4752.1^ns^ |

ns, not significantly different between genotypes under each water treatment and soil layer
